# Chemometric evaluation of inorganic and organic parameters found in *Rosaceae* plants proposed as food supplements

**DOI:** 10.1016/j.fochx.2024.101248

**Published:** 2024-02-28

**Authors:** Michaela Zeiner, Iva Juranović Cindrić, Ivan Nemet, Ivana Šola, Heidelore Fiedler

**Affiliations:** a Man-Technology-Environment Research Centre, School of Science and Technology, Örebro University, Fakultetsgatan, 1 70182 Örebro, Sweden; b Department of Chemistry, Faculty of Science, University of Zagreb, Horvatovac 102a, 10000 Zagreb, Croatia; c Department of Biology, Faculty of Science, University of Zagreb, Horvatovac 102a, 10000 Zagreb, Croatia

**Keywords:** Bioactive compounds, Chemometric analysis, Elemental pattern, Genus-specific evaluation, Food supplement, *Rosaceae* inflorescences

## Abstract

This study discusses the organic and inorganic composition of young inflorescence tissues of seven medical plants from the *Prunus*, *Malus*, and *Chaenomeles* families. These plants contain bioactive compounds with antioxidant and cytotoxic properties, and the study determined 29 elements, including essential and potentially harmful ones, established correlations with inorganic and organic compounds, as well as antioxidative and cytotoxic effects. The elemental patterns show that the plants contribute beneficial essential elements to the human diet. The levels of toxic elements in the plants are within safe limits set by the World Health Organization for medicinal herbs. The results confirmed genus- and species-specific uptake and accumulation. Positive correlations between d-block metals and alkaline earth metals in the inflorescences were found alongside statistically significant differences between analyte categories regarding macro-, micro- and trace elements and bioactive compounds. These correlations need to be considered when giving dietary recommendations or advice for uses as home-remedies.

## Highlights

• *Rosaceae* inflorescences contain essential elements beneficial to human diet.
• Toxic elements are present in contents lower than the limit values.
• Genera and species showed specific uptake and accumulation pattern.
• Content of d-block metals and alkaline earth metals were positively correlated.

## Introduction

Botanical gardens are specialized gardens that are dedicated to the cultivation, study, and display of a wide variety of plants and play a crucial role in scientific research. They provide a controlled environment for studying plant species, conducting experiments, and collecting data. Researchers can explore plant taxonomy, genetics, ecology, conservation, and chemical analysis within the garden's curated collections (Chen and Sun, 2018, Juranović-Cindrić et al., 2018).

The presence of essential as well as potentially toxic elements, including heavy metals, in the environment can be effectively assessed by examining the vegetation. Plants serve as reliable indicators due to their ability to absorb metals through root uptake and their exposure to metal deposition from both rainfall and dry deposition on their outer surfaces. This makes plants valuable tools for monitoring pollution over different spatial and temporal scales. Additionally, metals obtained from the soil through root uptake contribute to the metal content found in plant tissues (Wang and Zhang, 2020, Singh et al., 2022).

Metals play vital roles in plant physiology, and deficiencies in these elements are common in agricultural crops. Plants utilize metals as structural components in carbohydrates and proteins, as well as essential constituents of organic molecules crucial for normal metabolism (e.g., magnesium in chlorophyll and phosphorus in ATP). Metals also function as enzyme activators (e.g., potassium) and help maintain osmotic balance within plants (Soetan et al., 2010). For a *Brassicaceae* variety enhancement of flowering by elevated Ni supply was reported (Ghasemi et al., 2014).

The genetic background of plants does not only influence the uptake and accumulation of metals, but also the content of secondary metabolites such as flavonoids (Kaundun et al., 1997, Idžojtić et al., 2005). Studies have demonstrated correlation between metals and flavonoids, such as enhancement of the transportation of flavonoids within plants through their complexation with metals (Šola et al., 2015). Phenolic compounds provide protection against UV radiation, pathogens, and herbivores, and they play a significant role in normal plant growth and development (Kumar et al., 2020).

The contents of different bioactive compounds and the resultant antioxidant capacities make various plant species of interest for medical applications alongside usages as food supplements (Jamshidi-Kia et al., 2018). Regarding dietary flavonoids, it must be taken into account that the beneficial effects are not only due to their presence in a certain product, but also indirectly due to their effects on regulating gut microbiota which in turn leads to positive health effect (Li et al., 2023). Even if the beneficial properties are proven there might be a hidden health risk by the metal content of such products (Rai et al., 2019, Ćwieląg-Drabek et al., 2020).

Another reason for thorough characterisation of plant derived food (supplementary) material, is the increase of adulterated products. Food adulteration typically refers to the inclusion or exclusion of any substance in food, altering its standard composition and quality with the main goal of reducing production costs (Haji et al., 2023). The variability in climate, soil composition, and geographical region affects the phytochemical composition of herbal formulations, posing challenges to standardization. Adulteration and substitution of herbal drugs are increasing due to deforestation, compromising their safety and efficacy. The scarcity of genuine herbal drugs results from adulteration, substitution, and a lack of skilled personnel. Advanced quality control techniques and standards are essential to ensure the quality of medicinal herbal products. Standardization involves identifying, assessing physical, chemical, and biological properties to confirm the quality and purity of herbs, defining the freshness and overall quality of herbal products. (Balekundri & Mannur, 2020). Combined studies of inorganic and organic analytes leads to a deeper understanding of plants’ composition (Liu et al., 2019, Zeiner et al., 2023).

The present study focuses on the determination of the elemental composition of young inflorescences tissues of seven plants covering *Prunus, Malus* and *Chaenomeles*. They have been proven to possess biopotential through the content of bioactive compounds, different bioactive compounds, antioxidant, and cytotoxic capacities (Šola et al., 2022). The elements were selected in order to cover essential ones as well as potentially harmful ones, all together 29 were determined, namely Ag, Al, As, Ba, Be, Bi, Ca, Cd, Co, Cr, Cu, Fe, Ga, K, Li, Mg, Mn, Mo, Na, Ni, Pb, Rb, Se, Sr, Te, Tl, U, V, and Zn. Apart from the classical elemental characterisation, correlation calculations and chemometric evaluations were performed to prove the hypothesis that inorganic and organic compounds as well as elements and antioxidative and cytotoxic effects are correlated with each other.

## Material and methods

### Glassware and chemicals

Before use, all glassware and plasticware underwent cleaning with a diluted solution of nitric acid (7 mol/L). Ultrapure water with a resistivity greater than 18 MΩ∙cm was generated using an in-house apparatus.

High-quality reagents were employed for the analysis. Nitric acid and hydrochloric acid of p.a. (pro analysis) grade were provided by Merck (Germany). To prepare the calibration standards, a multi-element standard (ICP Multielement Standard IV; 1000 mg/L) from Merck (Germany) served as the stock solution. For method validation purposes, a certified reference material consisting of strawberry leaves was procured from LGC Standards (United Kingdom; LGC7162).

### Sample description and pretreatment

Rosaceae inflorescence samples from seven different varieties, namely *Prunus avium* (Pa), *Prunus serrulata* (Ps), *Prunus serrulate ‘Kiku Shidare Zakura’* (PsKss), *Prunus yedoensis* (Py), *Malus purpurea* (Mp), *Malus floribunda* (Mf), *Chaenomeles japonica* (Cj) were individually collected three times in April 2018 at the Botanical Garden of the Faculty of Science, University of Zagreb, Croatia. The collected plant material (inflorescenses) was subjected to freeze-drying using an Alpha 1–2 Christ freeze-dryer. Afterward, the dried material was pulverized using a pestle and mortar. (Šola et al., 2022) The resulting powders were utilized for elemental analysis.

The samples underwent elemental analysis using inductively coupled plasma mass spectrometry (ICP-MS) following a prior step of microwave-assisted digestion. For the decomposition of the plant tissue, a combination of nitric acid and hydrogen peroxide was employed. In triplicates, 0.1 g to 0.3 g of pulverised sample was weighed to the nearest 0.01 mg and mixed with 6 mL of nitric acid (50:50 *v/v*) and 2 mL of hydrogen peroxide (30 %) in a Teflon digestion vessel. Subsequently, the vessels were put into a Microwave System (MWS-2 Speedwave Berghof) where the following temperature program was applied: 20 min at 110 °C (600 W), 20 min at 170 °C (750 W), and 20 min at 120 °C (500 W). After the digestion process, the clear solutions were allowed to cool to room temperature and then transferred to 10 mL-volumetric flasks and filled up with deionized water. Prior to measurement all digest solutions were diluted 1:10 with ultrapure water.

### ICP-MS measurements

The applied analytical method has been validated in a previous study (Zeiner et al., 2022, Zeiner et al., 2022), the key data are summarized here. Metal concentrations in the solutions were determined using an inductively coupled mass spectrometry system (ICP-MS Agilent 7500cx, Tokyo, Japan) under the following experimental conditions: output power of 1500 W, 15 L/min Ar for the plasma, 0.9 L/min Ar as auxiliary gas, and 0.2 L/min Ar as nebulizer gas. A MicroMist nebulizer was employed, with a sample flow rate of 0.3 mL/min. After nebulization, the sample passed through a Scott double-pass spray chamber.

The analysis focused on the following elements, listed in order of increasing *m*/*z* (mass-to-charge ratio): ^7^Li, ^9^Be, ^23^Na, ^24^Mg, ^27^Al, ^39^K, ^43^Ca, ^51^V, ^53^Cr, ^55^Mn, ^56^Fe, ^59^Co, ^60^Ni, ^63^Cu, ^66^Zn, ^69^Ga, ^75^As, ^82^Se, ^85^Rb, ^88^Sr, ^95^Mo, ^107^Ag, ^111^Cd, ^125^Te, ^137^Ba, ^205^Tl, ^204+206+207+208^Pb, ^209^Bi, and ^238^U. For K, V, Cr, Fe, Cu, As, and Se, the collision cell was activated with a helium flow rate of 5 mL/min to minimize interferences.

Quantification of the 29 selected elements was achieved using five- or six-point external calibration curves, the calibration ranges were adjusted according to the expected concentration levels of each analyte. To compensate for non-spectral interferences, rhodium (^103^Rh) was added as an internal standard to all test solutions, including standard solutions, blanks, reference materials, and samples, at a final mass concentration of 10 µg/L. All measurements were performed in triplicate.

### Chemometric evaluation

All data were handled using Microsoft Office 365 Excel® (Microsoft corporation, 2018). R packages (versions 4.0.3 and 4.0.5) (R Core Team R, 2023) were applied for further statistical evaluations and visualization as described below. Similar approaches for evaluating such datasets can be found in the literature (Pan et al., 2022).

After conducting a normality test using histogram and density tests, it was determined that the samples did not follow a normal distribution. Consequently, non-parametric testing was employed using the Kruskal-Wallis H test to examine whether there were statistically significant differences between the independent variables and dependent variables. For post-hoc analysis, the pairwise Wilcoxon test was performed. The p-values were adjusted using the Benjamini-Hochberg method, and a significance level of p = 0.05 was set.

To assess the correlation between variables, the Pearson method was utilized, and a heatmap with hierarchical clustering was constructed using Euclidean distances and the Ward method. Multivariate methods, such as hierarchical cluster analysis (HCA) and principal component analysis (PCA), were applied to evaluate similarities, correlations, or differences between datasets and metadata.

HCA was employed to identify natural groupings (clusters) within the dataset, aiming to reduce variation (increase similarity) within each group and maximize variation (decrease similarity) between the groups. Clustering was performed using Euclidean distances and the Ward method, which minimizes variance within clusters.

PCA, a multivariate projection method, was used to extract and display systematic variation in the form of new variables that are linear combinations of the original variables. The principal component represents the maximum variation (Fig. 4, Fig. 5).

The chemometric evaluation covers the analytes from the present study, but includes also the following parameters for their biopotential, namely total phenolics (TP), total flavonoids (TF), total nonflavonoids (TNF), total tannins (TT), condensed tannins (CT), soluble sugars (SS), gallic acid, caffeic acid, the antioxidant capacity (ABTS, FRAP and DPPH), alongside their in vitro anti-proliferative effect on human hepatocellular (HepG2), colorectal (HCT 116) carcinoma cells, and non-tumorigenic skin keratinocytes (HaCaT). These parameters have been determined in a previous study to characterise underutilised *Rosaceae* inflorescences (Šola et al., 2022). All analytes have been categorized as shown in Table 1.

**Table 1:** Analytes and corresponding categories.

**Analyte**	**Category**	**Analyte**	**Category**
HepG2	Carcinog	Na	macro
HCT_116	Carcinog	Mg	macro
HaCaT	Carcinog	Al	macro
ABTS	Antioxidant	K	macro
FRAP	Antioxidant	Ca	macro
DPPH	Antioxidant	Fe	macro
TP	Biopotential	Cr	micro
TF	Biopotential	Mn	micro
TNF	Biopotential	Ni	micro
TT	Biopotential	Cu	micro
CT	Biopotential	Zn	micro
SS	Biopotential	Ba	micro
Gallic acid	Biopotential	Li	trace
Caffeic acid	Biopotential	V	trace
Be	NC	Co	trace
Rb	NC	Ga	trace
Sr	NC	Se	trace
Ag	NC	As	toxic
U	NC	Mo	toxic
		Cd	toxic
		Pb	toxic

## Results and discussion

### Elemental content in the *Rosaceae* inflorescences

**Fig. 1:**
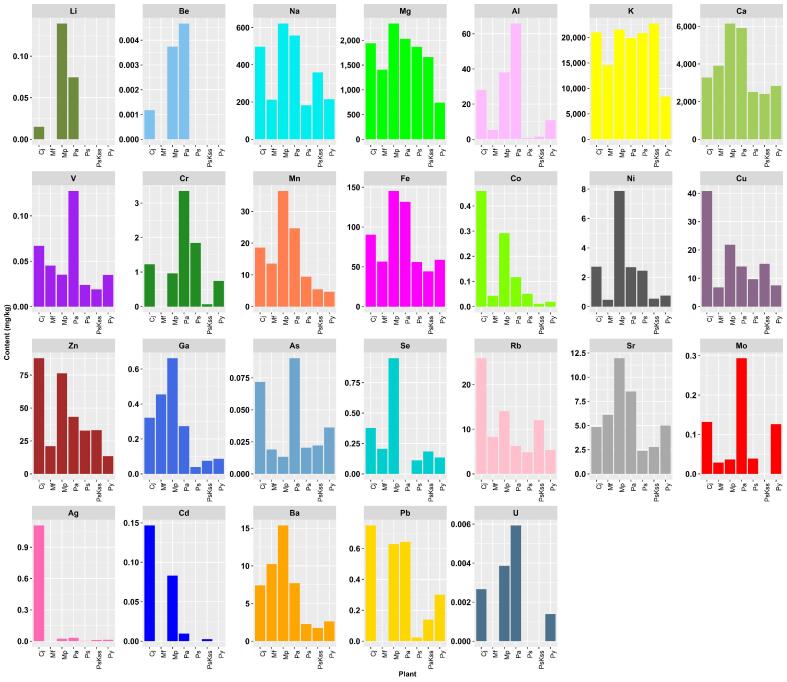
Bar graph of mean mass fraction of each detected analyte in mg/kg.

Multiple digests of seven plant samples were analyzed for the 29 elements given above. Out of those, Tl, Te, and Bi could not be detected in any sample. Li and Be were only found in Pa, Mp, and Cj. Cd and U could be quantified in four different species, the former in Pa, PsKss, Mp and Cj, the latter in Pa, Py, Mp and Cj. Cr, Se, Mo, Ag, and Pb were found in all except one sample, namely not in Mf, Pa, PsKss, and the last two not in Mf, respectively. 17 analytes, namely Na, Mg, Al, K, Ca, V, Mn, Fe, Co, Cu, Zn, Ga, As, Rb, Sr, and Ba were present in all analysed plant samples. The four macro elements were present in the order Ca > K > Mg > Na. The mean mass fractions of all elements quantified are shown in the bar graph in Fig. 1, clear differences in their pattern can be seen. Higher variation in the elemental content between species were found for micro- and trace elements compared to macro elements. Even when grown under identical conditions (same soil, same climatic situation, same location, same harvesting time), the uptake and accumulation patterns vary strongly between plant types and species. This effect has been reported also for trees (e.g. pine species) and different medical herbs (Zeiner et al., 2015, Zeiner et al., 2017, Bzour et al., 2017, Juranović Cindrić et al., 2019). By collecting all plant samples from the same growing site (i.e. the botanical garden) ensured that atmospheric deposition as well as influence of soil composition as influencing parameters for elemental uptake can be minimized. Even few meters in altitude might have a measurable impact on elemental content in leaves and flowers for herbs grown in urban area (Martini et al., 2023). Comparison with other edible inflorescences (banana heart – *Musa paradisiaca*) shows similar values for the macroelements Mg (1716 mg/kg) and Ca (2854 mg/kg), but unexpectedly neither Na nor K was present in detectable amounts (Rosa et al., 2020). Regarding microelements, the contents found for Cu, Ni, Cr, Zn, and Fe (Rosa et al., 2020) are all much less than those obtained in the present study for the *Rosaceae* inflorescences. Due to the usage of the analysed plants mainly because of their organic composition, which is reported in literature in most cases for the fruits (Nunes et al., 2021), there is a lack of comparison studies for the elemental composition. A study by Plessi and colleagues analysed eight metals in *P. avium* fruits (Plessi et al., 1998). Their results for the contents of Zn, Fe, Mn, Na, K, Mg, Ca are in the same order of magnitude, but the differences in sample type (plant part), harvesting time, growing do not allow for proper comparison. Thus, every species needs to be thoroughly analysed to established reference data, which can be further used for adulteration detection or risk assessments. The individual elemental composition has been also reported for different pepper species as basis for characterising food stuff (Zeiner et al., 2023). Furthermore, the elemental pattern can give hint of geographical origin of plant derived food products (Zeiner et al., 2022, Zeiner et al., 2022).

In order to minimize potential harmful effects on humans by certain elements, limits values for medicinal herbs are given by different regulations (Inada et al., 2023). All seven analysed plant samples contain As and Cd below the stated maximum values. Lead exceeds the limit of 0.3 mg/kg given by International Council for Harmonisation of Technical Requirements for Pharmaceuticals for Human Use in Pa, Mp, and Cj (Inada et al., 2023). However, these plants would be acceptable based on the World Health Organisation guideline which sets 10 mg/kg as upper limit (Inada et al., 2023).

**Fig. 2:**
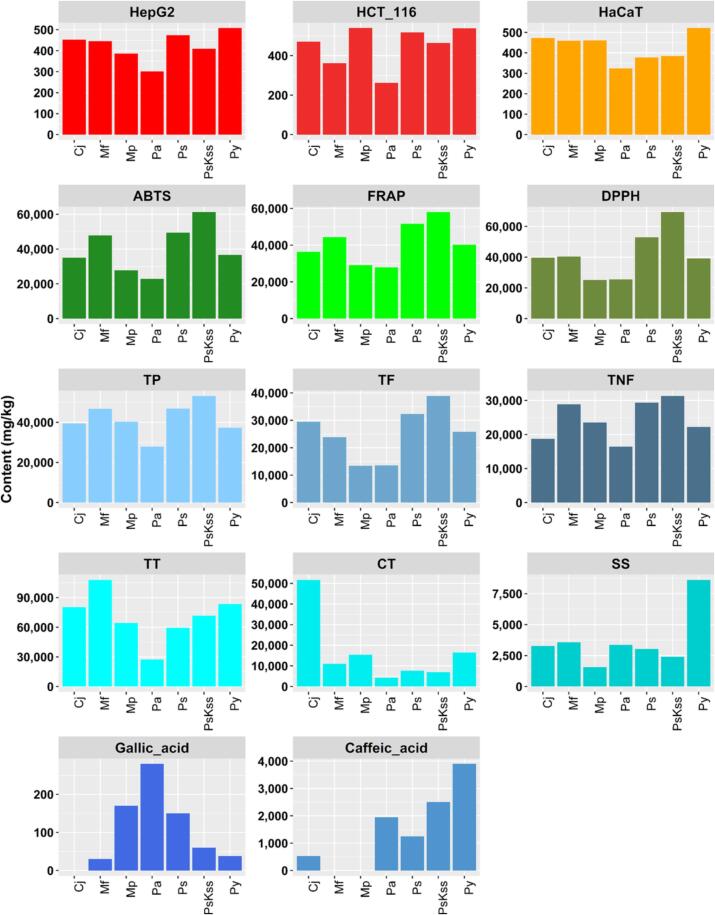
Bar graph of organics parameters. Row 1: carcinog expressed as IC50 in µg/mL; rows 2–4: antioxidant and biopotential as mass fraction in mg/kg (Šola et al., 2022).

For comparison reasons the organic compounds as well as the determined bioactive ingredients of the samples are shown in Fig. 2 as bar graph. Whilst the values differ a lot for the organic substances between different varieties, the anti-proliferative activity is more evenly distributed.

### Chemometric evaluation

Tables containing the descriptive statistics for the samples and parameters grouped according to the genus (*Chaenomeles - C, Malus - M, Prunus - P*) are contained in the supplementary information as Table S1 and for the analyte categories in Table S2.

**Fig. 3:**
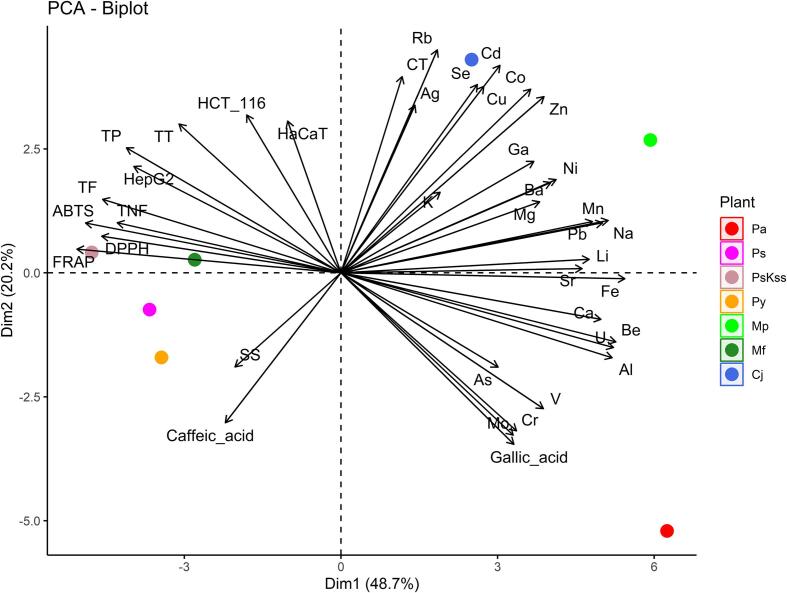
PCA biplot with loading plot (analytes) and score plot (plant).

**Fig. 4:**
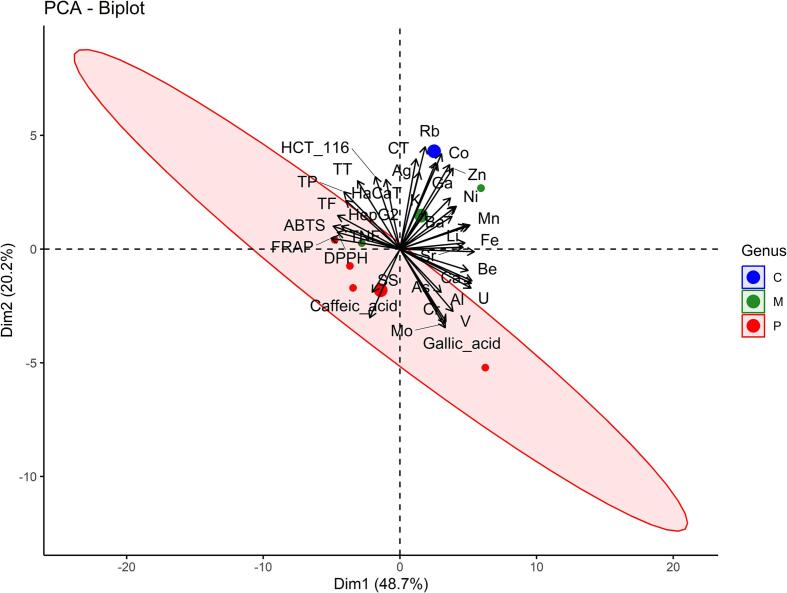
PCA biplot with ellipses around genus.

**Fig. 5:**
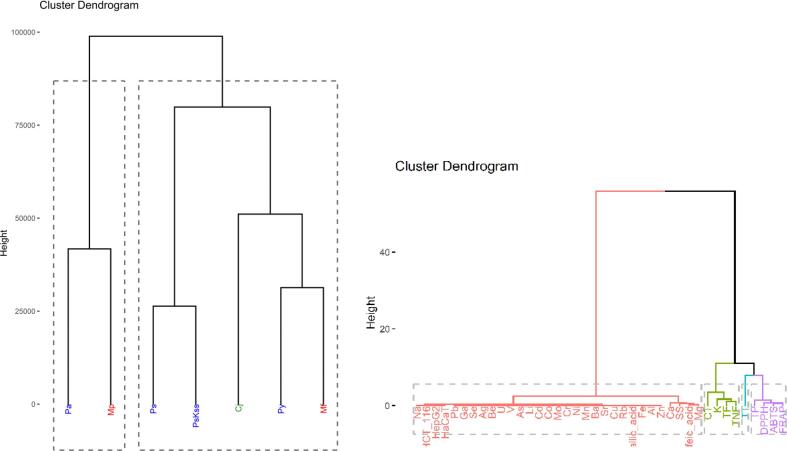
Dendrograms of hierarchical cluster analysis. Above: colored by plant; below: three clusters colored by branches in the dendrogram and analytes on x-axis.

Whereas all single elemental contents to do not show the same pattern for the different genera, the PCA biplots (Figs. 3 and 4), based on all variables, shows a grouping, the found similarities being also expressed in the dendrograms (Fig. 5).

*Chaenomeles* and *Malus* species are located mainly in the first and second quartiles, in contrast to *Prunus* species which are all except one in the lower part of the biplot, namely in the third and fourth quartile. The PCA explained approx. 69 % of the total variation among the samples, with PC1 accounting for 48.7 % of the variance and PC2 accounting for 20.2 %. The variables contributing to PC1 above average are Fe > Be > U > Al > Mn > FRAP > Na > Ca > ABTS > Pb > Li > Sr > DPPH > TF > TNF > Ni > TP > Ba > HepG2 > Zn > V, and below average Mg > Ga > Co > Cr > gallic acid > Mo > TT > Cd > As > Cu > Se > caffeic acid > SS > K > Rb > HCT116 > Ag > CT > HaCat. On the other hand, PC2 is determined by Rb > Cd > CT > Se > Cu > Co > Zn > gallic acid > Ag > Mo > Cr > HCT116 > HaCat > caffeic acid > TT > V > TP (mean contribution) > Ga > HepG2 > As > SS > Ni > Ba > Al > K > U > TF > Mg > Be > Mn > Pb > Na > TNF > ABTS > Ca > DPPH > FRAP > Li > Fe > Sr. High positive correlation can be seen for antioxidative activity (ABTS, FRAP and DPPH), in contrast to the in vitro anti-proliferative effect where only two, namely HCT 116 and HaCaT are highly correlated.

**Fig. 6:**
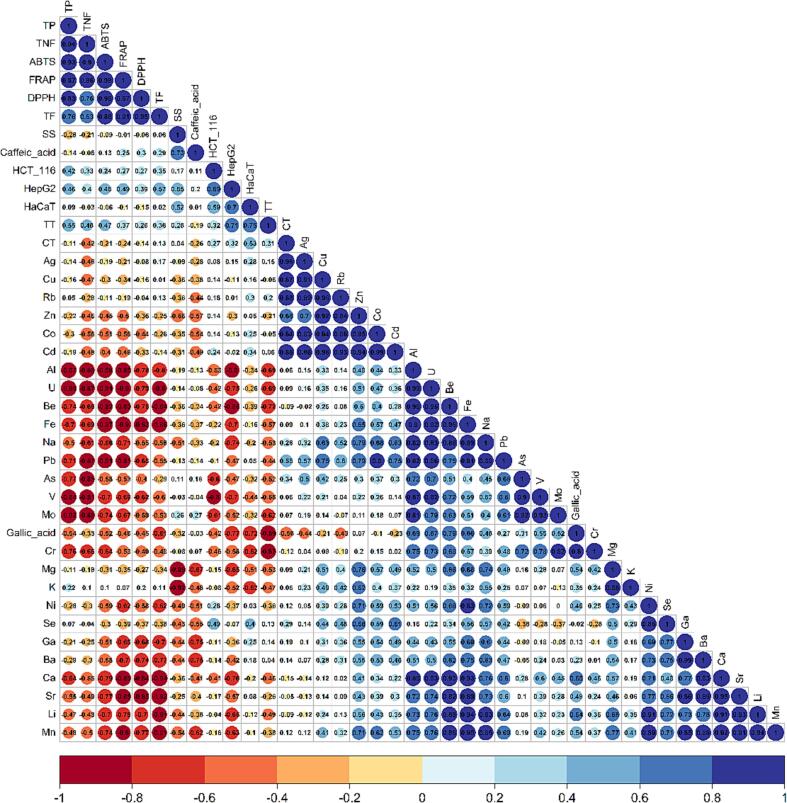
Pearson correlation between all quantified elements and biopotential describing parameters.

**Fig. 7:**
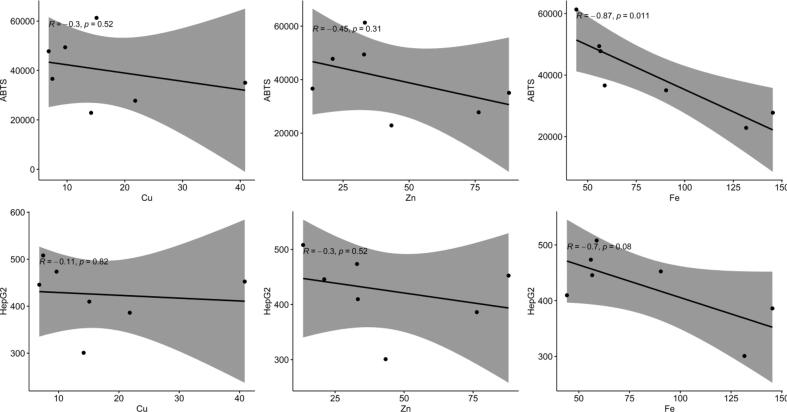
Correlation graphs for Cu, Zn, and Fe with ABTS and HepG2, resp.

A deeper insight into the correlation between all single variables to prove the former stated hypothesis, can be seen in Fig. 6 which shows the Spearman correlation between all parameters. Also, there the correlation between organic compounds to each other is obvious and higher than between elements and biopotential variables. Regarding the metals, Ba, Ca, Sr (group II in the periodic table) are highly correlated. Furthermore, the d-block elements Ag, Cu, Zn, Co, Cd are closely related. Zn is a metal which can cause oxidative stress, but also act as antioxidant, even if essential for humans, higher concentrations can be toxic to intestine cells (Zödl et al., 2003a). Looking at the correlation chart, no close relation between the antioxidative potential and Zn nor between this metal and the anti-proliferative activity parameters was found. Cu, which might also be toxic to intestine cells due to the ability to catalyze the formation of free radicals (Zödl et al., 2003b), shows like Zn no correlation with ABTS, FRAP, DPPH or anti-proliferative effects. Conversely Fe, also known as being toxic to intestine cells through generating reactive oxygen species (Zödl et al., 2004), shows strong negative correlation to ABTS, FRAP, DPPH, and HepG2. Correlation graphs for Cu, Zn, and Fe with ABTS and HepG2, resp. are shown in Fig. 7.

#### Transformed dataset

Table S2 and Fig. S1 show that the sum of the in vitro anti-proliferative effects and the three antioxidants were similar in all seven samples. Large differences for the sums were seen for the biopotential with TT dominating (sum ∼ 500 000 mg/kg) followed by TP (sum ∼ 300 000 mg/kg). Also, for the macro, micro, trace, toxic, and the not classified elements, there was always one element dominating: K within the macro elements, Zn within the microelements, Pb within the toxic, and Rb within the not classified elements. Only for the trace elements, within a small range from < 0.3 mg/kg to 2 mg/kg, two elements, namely Se and Ga, were dominating over Co ≫ V > Li. For the trace elements, the two *M* samples had largest contribution whereas for all other analyte categories, these two samples had minor impact.

**Fig. 8:**
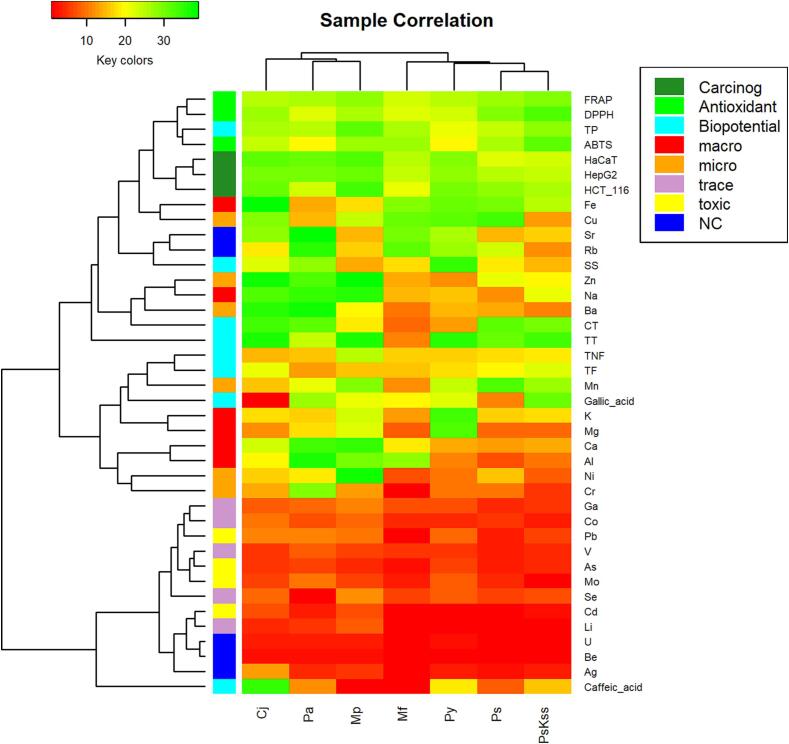
Heatmap with the samples in columns and the organic chemicals and elements in rows, coloured according to the analyte category.

But within between the samples, quite large differences can be seen (Fig. S2). Note: always one outlier for macro elements (micro as well except three). The heatmap is presented in Fig. 8 and the corresponding biplot in Fig. S3.

## Conclusions

The present study continued the characterisation of inflorescences of seven *Rosaceae* varieties coming from three genera, which have been investigated for their phytochemical profile, in vitro antioxidant activity, and antidiabetic, anti-inflammatory and antiproliferative potential previously. The determined elemental patterns prove their beneficial contribution to human diet through their content of essential elements and on the other hand no health risks are to be expected by toxic elements, since their contents are below the maximum values for medicinal herbs stated by WHO. By keeping the growing conditions the same for all plants, species specific uptake and accumulation patterns could be verified. Positive correlations were found between d-block metals as well as between alkaline earth metals.

Analysis of variance, using the Kruskal Wallis test showed that the analyte categories were significantly different (p ≤ 2.2 × 10^-16^). The pairwise assessment however showed that trace and toxic elements in the samples were not statistically significantly different (p = 0.372) and both not different from the NC elements (p = 0.981 and p = 0.676, resp.). Also, the samples could not be differentiated as for content of microelements and Carcinog parameters (p = 0.410). Thus, when giving dietary recommendations for essential elements with high nutritional importance care should be taken since negative anti-carcinogenic effects may be associated.

A thorough characterisation of plant derived food products is the basis for adulteration detection. This paper is one step towards this goal.

## CRediT authorship contribution statement

**Michaela Zeiner:** Writing – original draft, Methodology, Formal analysis, Data curation, Conceptualization. **Iva Juranović Cindrić:** Methodology. **Ivan Nemet:** Methodology. **Ivana Šola:** Methodology. **Heidelore Fiedler:** Writing – original draft, Visualization.

## Declaration of competing interest

The authors declare that they have no known competing financial interests or personal relationships that could have appeared to influence the work reported in this paper.

## Data Availability

Data will be made available on request.
