# Supplementary material for: Chemometric evaluation of inorganic and organic parameters found in Rosaceae plants proposed as food supplements
Source: Food Chem X. 2024 Feb 28;22:101248. doi: 10.1016/j.fochx.2024.101248 (PMC10912348; doi:10.1016/j.fochx.2024.101248)
Supplement: Supplementary data 1 [file mmc1.docx]

**Supplementary information**:

Table S1: Descriptive statistics by Genus

|  | **C (n = 1)** | **M (n = 2)** | **P (n = 4)** | **Overall (n = 7)** |
| --- | --- | --- | --- | --- |
| **HepG2** | | | | |
| Mean (SD) | 452 (NA) | 416 (42.1) | 423 (91.1) | 425 (67.8) |
| Median [Min, Max] | 452 [452, 452] | 416 [386, 446] | 442 [301, 508] | 446 [301, 508] |
| **HCT_116** | | | | |
| Mean (SD) | 471 (NA) | 451 (126) | 445 (126) | 450 (103) |
| Median [Min, Max] | 471 [471, 471] | 451 [362, 540] | 491 [262, 538] | 471 [262, 540] |
| **HaCaT** | | | | |
| Mean (SD) | 473 (NA) | 460 (1.49) | 402 (84.3) | 429 (68.5) |
| Median [Min, Max] | 473 [473, 473] | 460 [459, 461] | 381 [324, 522] | 459 [324, 522] |
| **ABTS** | | | | |
| Mean (SD) | 35100 (NA) | 37800 (14100) | 42600 (16600) | 40100 (13400) |
| Median [Min, Max] | 35100 [35100, 35100] | 37800 [27800, 47800] | 43000 [22900, 61300] | 36600 [22900, 61300] |
| **FRAP** | | | | |
| Mean (SD) | 36400 (NA) | 36700 (10800) | 44500 (13300) | 41100 (11200) |
| Median [Min, Max] | 36400 [36400, 36400] | 36700 [29100, 44400] | 46000 [27900, 58100] | 40300 [27900, 58100] |
| **DPPH** | | | | |
| Mean (SD) | 39600 (NA) | 32700 (10800) | 46800 (18800) | 41700 (15500) |
| Median [Min, Max] | 39600 [39600, 39600] | 32700 [25100, 40400] | 46100 [25500, 69400] | 39600 [25100, 69400] |
| **TP** | | | | |
| Mean (SD) | 39400 (NA) | 43500 (4560) | 41300 (11100) | 41700 (8180) |
| Median [Min, Max] | 39400 [39400, 39400] | 43500 [40300, 46700] | 42100 [27800, 53100] | 40300 [27800, 53100] |
| **TF** | | | | |
| Mean (SD) | 29500 (NA) | 18600 (7350) | 27600 (10800) | 25300 (9420) |
| Median [Min, Max] | 29500 [29500, 29500] | 18600 [13400, 23800] | 29100 [13600, 38900] | 25800 [13400, 38900] |
| **TNF** | | | | |
| Mean (SD) | 18700 (NA) | 26200 (3770) | 24800 (6840) | 24300 (5680) |
| Median [Min, Max] | 18700 [18700, 18700] | 26200 [23500, 28900] | 25800 [16400, 31300] | 23500 [16400, 31300] |
| **TT** | | | | |
| Mean (SD) | 80300 (NA) | 86100 (30800) | 60500 (24200) | 70600 (24800) |
| Median [Min, Max] | 80300 [80300, 80300] | 86100 [64300, 108000] | 65500 [27300, 83600] | 71600 [27300, 108000] |
| **CT** | | | | |
| Mean (SD) | 51700 (NA) | 13200 (3160) | 8880 (5310) | 16200 (16300) |
| Median [Min, Max] | 51700 [51700, 51700] | 13200 [11000, 15500] | 7370 [4250, 16500] | 11000 [4250, 51700] |
| **SS** | | | | |
| Mean (SD) | 3270 (NA) | 2570 (1430) | 4360 (2860) | 3690 (2280) |
| Median [Min, Max] | 3270 [3270, 3270] | 2570 [1560, 3580] | 3210 [2410, 8610] | 3270 [1560, 8610] |
| **Gallic_acid** | | | | |
| Mean (SD) | 0 (NA) | 100 (99.0) | 132 (110) | 104 (100) |
| Median [Min, Max] | 0 [0, 0] | 100 [30.0, 170] | 105 [38.0, 280] | 60.0 [0, 280] |
| **Caffeic_acid** | | | | |
| Mean (SD) | 530 (NA) | 0 (0) | 2400 (1120) | 1450 (1440) |
| Median [Min, Max] | 530 [530, 530] | 0 [0, 0] | 2230 [1250, 3900] | 1250 [0, 3900] |
| **Li** | | | | |
| Mean (SD) | 0.0151 (NA) | 0.0696 (0.0984) | 0.0186 (0.0373) | 0.0327 (0.0543) |
| Median [Min, Max] | 0.0151 [0.0151, 0.0151] | 0.0696 [0, 0.139] | 0 [0, 0.0746] | 0 [0, 0.139] |
| **Be** | | | | |
| Mean (SD) | 0.00118 (NA) | 0.00187 (0.00264) | 0.00117 (0.00233) | 0.00137 (0.00200) |
| Median [Min, Max] | 0.00118 [0.00118, 0.00118] | 0.00187 [0, 0.00374] | 0 [0, 0.00467] | 0 [0, 0.00467] |
| **Na** | | | | |
| Mean (SD) | 498 (NA) | 417 (289) | 329 (171) | 378 (182) |
| Median [Min, Max] | 498 [498, 498] | 417 [213, 621] | 287 [183, 557] | 360 [183, 621] |
| **Mg** | | | | |
| Mean (SD) | 1940 (NA) | 1870 (660) | 1570 (576) | 1710 (518) |
| Median [Min, Max] | 1940 [1940, 1940] | 1870 [1400, 2340] | 1760 [740, 2030] | 1870 [740, 2340] |
| **Al** | | | | |
| Mean (SD) | 28.1 (NA) | 21.7 (23.1) | 19.7 (31.1) | 21.5 (24.1) |
| Median [Min, Max] | 28.1 [28.1, 28.1] | 21.7 [5.35, 38.1] | 6.22 [0.586, 65.8] | 10.9 [0.586, 65.8] |
| **K** | | | | |
| Mean (SD) | 21000 (NA) | 18100 (4950) | 18000 (6490) | 18400 (5140) |
| Median [Min, Max] | 21000 [21000, 21000] | 18100 [14600, 21600] | 20400 [8400, 22700] | 20900 [8400, 22700] |
| **Co** | | | | |
| Mean (SD) | 3290 (NA) | 5030 (1580) | 3420 (1670) | 3860 (1570) |
| Median [Min, Max] | 3290 [3290, 3290] | 5030 [3910, 6140] | 2680 [2410, 5910] | 3290 [2410, 6140] |
| **V** | | | | |
| Mean (SD) | 0.0669 (NA) | 0.0401 (0.00715) | 0.0513 (0.0512) | 0.0504 (0.0374) |
| Median [Min, Max] | 0.0669 [0.0669, 0.0669] | 0.0401 [0.0351, 0.0452] | 0.0294 [0.0190, 0.127] | 0.0351 [0.0190, 0.127] |
| **Cr** | | | | |
| Mean (SD) | 1.23 (NA) | 0.479 (0.678) | 1.50 (1.43) | 1.17 (1.16) |
| Median [Min, Max] | 1.23 [1.23, 1.23] | 0.479 [0, 0.958] | 1.29 [0.0698, 3.35] | 0.958 [0, 3.35] |
| **Mn** | | | | |
| Mean (SD) | 18.6 (NA) | 25.0 (16.2) | 11.0 (9.33) | 16.1 (11.5) |
| Median [Min, Max] | 18.6 [18.6, 18.6] | 25.0 [13.5, 36.5] | 7.39 [4.65, 24.7] | 13.5 [4.65, 36.5] |
| **Fe** | | | | |
| Mean (SD) | 90.4 (NA) | 101 (62.8) | 72.6 (39.8) | 83.3 (40.5) |
| Median [Min, Max] | 90.4 [90.4, 90.4] | 101 [56.6, 145] | 57.4 [44.2, 132] | 58.8 [44.2, 145] |
| **Co** | | | | |
| Mean (SD) | 0.460 (NA) | 0.168 (0.176) | 0.0495 (0.0483) | 0.142 (0.171) |
| Median [Min, Max] | 0.460 [0.460, 0.460] | 0.168 [0.0429, 0.292] | 0.0349 [0.0111, 0.117] | 0.0508 [0.0111, 0.460] |
| **Ni** | | | | |
| Mean (SD) | 2.72 (NA) | 4.17 (5.24) | 1.60 (1.11) | 2.50 (2.58) |
| Median [Min, Max] | 2.72 [2.72, 2.72] | 4.17 [0.463, 7.88] | 1.59 [0.542, 2.68] | 2.44 [0.463, 7.88] |
| **Cu** | | | | |
| Mean (SD) | 40.8 (NA) | 14.3 (10.6) | 11.6 (3.64) | 16.6 (11.9) |
| Median [Min, Max] | 40.8 [40.8, 40.8] | 14.3 [6.82, 21.8] | 11.9 [7.46, 15.1] | 14.2 [6.82, 40.8] |
| **Zn** | | | | |
| Mean (SD) | 87.8 (NA) | 48.7 (39.1) | 30.7 (12.5) | 44.0 (27.9) |
| Median [Min, Max] | 87.8 [87.8, 87.8] | 48.7 [21.0, 76.3] | 33.1 [13.4, 43.3] | 33.2 [13.4, 87.8] |
| **Ga** | | | | |
| Mean (SD) | 0.321 (NA) | 0.559 (0.146) | 0.118 (0.105) | 0.273 (0.229) |
| Median [Min, Max] | 0.321 [0.321, 0.321] | 0.559 [0.456, 0.662] | 0.0803 [0.0391, 0.273] | 0.273 [0.0391, 0.662] |
| **As** | | | | |
| Mean (SD) | 0.0717 (NA) | 0.0162 (0.00398) | 0.0423 (0.0328) | 0.0390 (0.0300) |
| Median [Min, Max] | 0.0717 [0.0717, 0.0717] | 0.0162 [0.0134, 0.0190] | 0.0292 [0.0205, 0.0903] | 0.0221 [0.0134, 0.0903] |
| **Se** | | | | |
| Mean (SD) | 0.377 (NA) | 0.579 (0.527) | 0.107 (0.0778) | 0.280 (0.317) |
| Median [Min, Max] | 0.377 [0.377, 0.377] | 0.579 [0.206, 0.951] | 0.122 [0, 0.184] | 0.184 [0, 0.951] |
| **Rb** | | | | |
| Mean (SD) | 25.9 (NA) | 11.2 (4.10) | 7.13 (3.32) | 11.0 (7.44) |
| Median [Min, Max] | 25.9 [25.9, 25.9] | 11.2 [8.29, 14.1] | 5.80 [4.87, 12.0] | 8.29 [4.87, 25.9] |
| **Sr** | | | | |
| Mean (SD) | 4.83 (NA) | 9.04 (4.13) | 4.67 (2.81) | 5.94 (3.36) |
| Median [Min, Max] | 4.83 [4.83, 4.83] | 9.04 [6.11, 12.0] | 3.89 [2.39, 8.51] | 5.01 [2.39, 12.0] |
| **Mo** | | | | |
| Mean (SD) | 0.132 (NA) | 0.0325 (0.00544) | 0.115 (0.130) | 0.0936 (0.102) |
| Median [Min, Max] | 0.132 [0.132, 0.132] | 0.0325 [0.0286, 0.0363] | 0.0824 [0, 0.294] | 0.0387 [0, 0.294] |
| **Ag** | | | | |
| Mean (SD) | 1.11 (NA) | 0.0118 (0.0167) | 0.0143 (0.0133) | 0.170 (0.414) |
| Median [Min, Max] | 1.11 [1.11, 1.11] | 0.0118 [0, 0.0236] | 0.0111 [0.00183, 0.0330] | 0.0121 [0, 1.11] |
| **Cd** | | | | |
| Mean (SD) | 0.147 (NA) | 0.0416 (0.0588) | 0.00305 (0.00457) | 0.0346 (0.0579) |
| Median [Min, Max] | 0.147 [0.147, 0.147] | 0.0416 [0, 0.0831] | 0.00126 [0, 0.00966] | 0.00252 [0, 0.147] |
| **Ba** | | | | |
| Mean (SD) | 7.41 (NA) | 12.8 (3.62) | 3.59 (2.77) | 6.76 (5.00) |
| Median [Min, Max] | 7.41 [7.41, 7.41] | 12.8 [10.2, 15.4] | 2.45 [1.74, 7.70] | 7.41 [1.74, 15.4] |
| **Pb** | | | | |
| Mean (SD) | 0.749 (NA) | 0.314 (0.444) | 0.277 (0.269) | 0.355 (0.316) |
| Median [Min, Max] | 0.749 [0.749, 0.749] | 0.314 [0, 0.628] | 0.220 [0.0237, 0.643] | 0.301 [0, 0.749] |
| **U** | | | | |
| Mean (SD) | 0.00266 (NA) | 0.00193 (0.00273) | 0.00183 (0.00281) | 0.00198 (0.00230) |
| Median [Min, Max] | 0.00266 [0.00266, 0.00266] | 0.00193 [0, 0.00386] | 0.000698 [0, 0.00593] | 0.00140 [0, 0.00593] |
| **P** | | | | |
| Mean (SD) | 0.749 (NA) | 0.314 (0.444) | 0.277 (0.269) | 0.355 (0.316) |
| Median [Min, Max] | 0.749 [0.749, 0.749] | 0.314 [0, 0.628] | 0.220 [0.0237, 0.643] | 0.301 [0, 0.749] |

Table S 2: Descriptive statistics by analyte category (transformed set)

|  | **Carcinog (n = 3)** | **Antioxidant (n = 3)** | **Biopotential (n = 8)** | **macro (n = 6)** | **micro (n = 6)** | **trace (n = 5)** | **toxic (n = 4)** | **NC (n = 5)** |
| --- | --- | --- | --- | --- | --- | --- | --- | --- |
| **Pa** | | | | | | | | |
| Mean (SD) | 296 (31.3) | 25400 (2520) | 11900 (11200) | 4770 (7750) | 16.0 (15.7) | 0.119 (0.100) | 0.259 (0.282) | 2.96 (4.12) |
| Median [Min, Max] | 301 [262, 324] | 25500 [22900, 27900] | 8910 [280, 27800] | 1290 [65.8, 19900] | 10.9 [2.68, 43.3] | 0.117 [0, 0.273] | 0.192 [0.010, 0.643] | 0.0330 [0.005, 8.51] |
| **Ps** | | | | | | | | |
| Mean (SD) | 456 (71.5) | 51300 (1790) | 22500 (22800) | 4250 (8210) | 9.76 (11.9) | 0.0448 (0.0412) | 0.0207 (0.0159) | 1.45 (2.17) |
| Median [Min, Max] | 474 [378, 517] | 51700 [49400, 53000] | 18500 [150, 59400] | 1020 [0.586, 20900] | 5.91 [1.85, 32.9] | 0.0391 [0, 0.110] | 0.0221 [0, 0.0387] | 0.00183 [0, 4.87] |
| **PsKss** | | | | | | | | |
| Mean (SD) | 420 (40.3) | 62900 (5850) | 25900 (27100) | 4530 (8960) | 9.35 (13.0) | 0.0578 (0.0763) | 0.0410 (0.0663) | 2.96 (5.21) |
| Median [Min, Max] | 410 [385, 464] | 61300 [58100, 69400] | 19200 [60.0, 71600] | 1010 [1.53, 22700] | 3.57 [0.0698, 33.2] | 0.0190 [0, 0.184] | 0.0123 [0, 0.139] | 0.0101 [0, 12.0] |
| **Py** | | | | | | | | |
| Mean (SD) | 523 (14.9) | 38700 (1880) | 24700 (26700) | 2040 (3290) | 4.93 (4.87) | 0.0549 (0.0548) | 0.116 (0.134) | 2.07 (2.83) |
| Median [Min, Max] | 522 [508, 538] | 39200 [36600, 40300] | 19400 [38.0, 83600] | 477 [10.9, 8400] | 3.63 [0.742, 13.4] | 0.0349 [0, 0.135] | 0.0812 [0, 0.301] | 0.0121 [0, 5.34] |
| **Mp** | | | | | | | | |
| Mean (SD) | 462 (76.7) | 27300 (2050) | 19800 (22600) | 5140 (8370) | 26.5 (27.3) | 0.416 (0.382) | 0.190 (0.293) | 5.21 (7.16) |
| Median [Min, Max] | 461 [386, 540] | 27800 [25100, 29100] | 14400 [0, 64300] | 1480 [38.1, 21600] | 18.6 [0.958, 76.3] | 0.292 [0.035, 0.951] | 0.060 [0.013, 0.628] | 0.024 [0.004, 14.1] |
| **Mf** | | | | | | | | |
| Mean (SD) | 422 (52.8) | 44200 (3700) | 27700 (36200) | 3360 (5700) | 8.68 (8.07) | 0.150 (0.188) | 0.0119 (0.0143) | 2.88 (4.02) |
| Median [Min, Max] | 446 [362, 459] | 44400 [40400, 47800] | 17400 [0, 108000] | 809 [5.35, 14600] | 8.53 [0, 21.0] | 0.0452 [0, 0.456] | 0.00951 [0, 0.0286] | 0 [0, 8.29] |
| **Cj** | | | | | | | | |
| Mean (SD) | 465 (11.3) | 37000 (2350) | 27900 (28400) | 4470 (8190) | 26.4 (33.5) | 0.248 (0.196) | 0.275 (0.318) | 6.37 (11.1) |
| Median [Min, Max] | 471 [452, 473] | 36400 [35100, 39600] | 24100 [0, 80300] | 1220 [28.1, 21000] | 13.0 [1.23, 87.8] | 0.321 [0.015, 0.460] | 0.139 [0.072, 0.749] | 1.11 [0.0012, 25.9] |





Figure S1: Stacked barplots by analyte and grouped by category





Figure S2: Box whisker (log scale above) for samples grouped by category
Units on y-axis: carcinog expressed as IC50 in µg/mL; all others as mass fraction in mg/kg


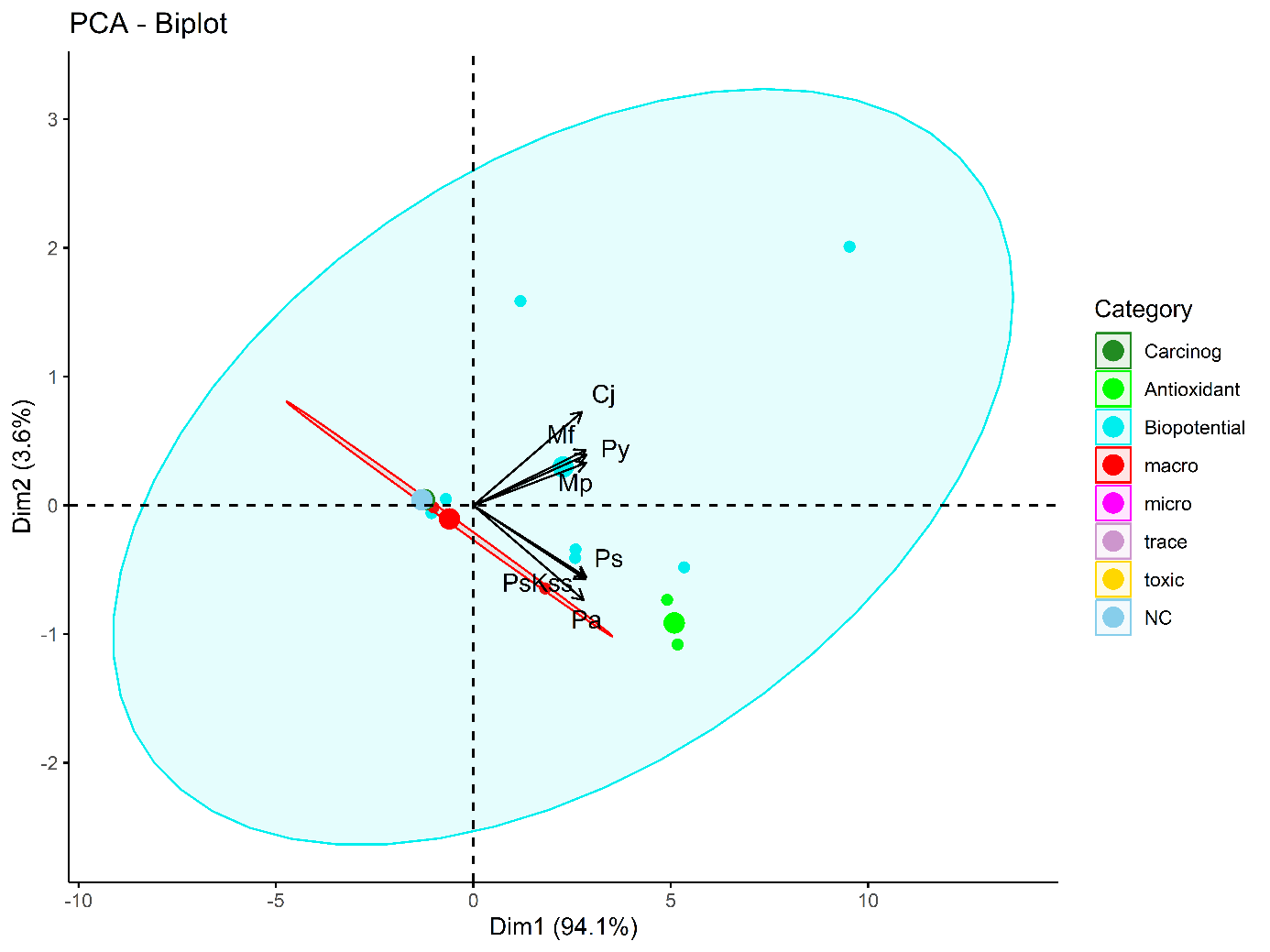


Figure S3: PCA for transformed data set with ellipse around Category
